# Validation of Stainless-Steel CHS Columns Finite Element Models

**DOI:** 10.3390/ma14071785

**Published:** 2021-04-04

**Authors:** Daniel Jindra, Zdeněk Kala, Jiří Kala

**Affiliations:** Institute of Structural Mechanics, Faculty of Civil Engineering, Brno University of Technology, Veveří 331/95, 60200 Brno, Czech Republic; kala.z@fce.vutbr.cz (Z.K.); kala.j@fce.vutbr.cz (J.K.)

**Keywords:** stainless steel, finite element numerical model, numerical model validation, Ramberg and Osgood model, CHS column buckling

## Abstract

Stainless-steel elements are increasingly used in a wide range of load-bearing structures due to their strength, minimal maintenance requirements, and aesthetic appearance. Their response differs from standard steels; therefore, it is necessary to choose a different procedure when creating a correct computational model. Seven groups of numerical models differing in the used formulation of elements integration, mesh density localization, nonlinear material model, and initial geometric imperfection were calibrated. The results of these advanced simulations were validated with published results obtained by an extensive experimental approach on circular hollow sections columns. With regard to the different slenderness of the cross-sections, the influence of the initial imperfection in the form of global and local loss of stability on the response was studied. Responses of all models were validated by comparing the averaged normalized ultimate loads and the averaged normalized deflections with experimentally obtained results.

## 1. Introduction

In comparison with the most commonly used carbon steel material, stainless steel is more recent in the field of structural engineering. For example, the UK’s first stainless-steel road bridge over the River Eamont in the village named Pooley Bridge was officially opened in October 2020. The first composite stainless-steel vehicular bridge in Europe (most probably also worldwide) was built in Cala Galdana on the island of Menorca, Spain, and was opened in June 2005 [1]. This duplex stainless-steel arch bridge over Algendar Creek replaced the existing reinforced concrete bridge which suffered severe corrosion issues due to the marine environment. Despite the higher cost of the stainless-steel material, the usage of this material is most beneficial in cases of structures with heavy traffic volumes or those exposed to such aggressive environment as was discussed for the case of the steel ASTM A1010 in a recent cost-efficiency study by Daghas et al. [2]. Usage possibilities of the stainless-steel material in combination with different materials have been recently studied by Pauletta et al. [3], who investigated the bond-slip behavior between stainless-steel reinforcement bars and concrete, or by Corradi et al. [4], who reviewed the use of stainless-steel profiles to reinforce or repair historical wooden structures. Research is also conducted also on the enhancement of the stainless-steel corrosion resistance by Dinu et al. [5], who proposed a certain improvement of the grade AISI 304 stainless steel.

In addition to higher cost and strong corrosion resistance, another significant difference, compared to ordinary carbon steel, from the aspect of mechanical properties is the nonlinear stress-strain behavior. The stress-strain curve of a stainless-steel material possesses no pronounced yield plateau and exhibits an early declination from the linear elastic behavior with significant strain hardening. Along with higher strength and higher ductility, these are the most significant differences compared to carbon steel [6]. Various analytical equations describing this material behavior are found in the literature, e.g., in the study by Hradil et al. [7]. A constitutive model of stainless steel in a high-strain range was investigated by Peng et al. [8]. The stress-strain curves were also compared by Real et al. [9]. These descriptions are based on the expressions originally proposed by Ramberg and Osgood [10], subsequently modified by Hill [11]. The use of 0.2% proof stress, as the equivalent of the yield stress, has become a standard industry practice [12]. The variability of material parameter values is significantly high among different stainless-steel grades and different groups (types according to metallurgical structure) [13]. The most commonly used types of stainless steel are austenitic, ferritic, and duplex ones.

Material parameters can be feasibly determined through the process of parameter identification, where either the analytical stress-strain curve or the stress-strain curve as the result yielded from the finite element numerical analysis is fitted to be in the best possible match with the experimentally measured data. Such material model calibration has been conducted by Jindra et al. [14]. Values of the material parameters could also be obtained, based on the informative Annex C of the European standard EN 1993-1-4 [15], from the tables in AS/NZS 4673 [16] or SEI/ASCE-8 [17]. Recently, a statistical study of not only stainless-steel material parameter values, based on the results consolidated over the last decades, has been conducted by Arrayago et al. [18].

The design guidance to determine the flexural buckling capacity of stainless-steel CHS (circular hollow section) members is provided in EN 1993-1-4 [15]. This approach is consistent with the same task for carbon steel structural elements described in EN 1993-1-1 [19]. Many existing data points (based on either physical experiments or properly validated geometrically and materially nonlinear finite element analyses with imperfections -GMNIA) lie below the current EN 1993-1-4 [15] flexural buckling curve, as described by Young et al. [20], Rasmussen et al. [21], Ashraf et al. [22], Theofanous et al. [23], or Shu et al. [24]. Originally, due to the insufficient amount of stainless-steel CHS experimental data at the EN standard creation time, the buckling curve was calibrated mainly by considering cold-formed rectangular hollow section (RHS) and square hollow section (SHS) results of the column buckling tests [21]. The buckling curve based on cold-formed RHS and SHS experimental results may not be appropriate for CHS due to increased material strength in hardened corner areas of RHS and SHS cross-sections [25].

Physical experiments on stainless-steel CHS elements exposed to compressive loading have been conducted by Rasmussen et al. [26], who investigated austenitic stainless-steel stub columns. Authors of similar research include Burgan et al. [27], Talja [28], Rasmussen [29], Young et al. [20], Uy et al. [30], Zhao et al. [31], Lam et al. [32], Gardner et al. [33], Bardi et al. [34], and Paquette et al. [35]. A wide range of loading eccentricities in the compression of CHS columns was investigated by Buchanan et al. [36]. It can be noted that the present article studies the static resistance of the column as a supporting structural member, and the heat and flow load cases [37] inside the tube are not considered.

The benchmark data presented in this study are obtained from a comprehensive experimental program conducted at Imperial College London (ICL) and Universitat Politècnica de Catalunya (UPC) and a previous research conducted by Buchanan et al. [38]. Tests of the two austenitic (A) cross-sections, 104 × 2 CHS and 106 × 3 CHS, and the duplex (D) cross-section 88.9 × 2.6 CHS were undertaken at ICL, and those of the two ferritic (F) cross-sections, 101.6 × 1.5 CHS and 80 × 1.5 CHS, were carried out at UPC [38].

To conduct further geometrically and materially nonlinear analyses of CHS stainless-steel columns in compression, the parametrical numerical finite element models created in ANSYS Classic(v 19.0.) utilizing APDL (ANSYS parametric design language) [39] need to be properly validated.

This contribution aims at the validation of the numerical FE models of CHS columns buckling tests and comparison of the validation results with the research of Mr. Buchanan, Mrs. Esther Real, and Mr. Leroy Gardner [38], where another FE software, Abaqus/CAE 2016 [40], was used, and a slightly different mesh size [38] was used. Moreover, this study compares the results of various modeling approaches, where either different mesh sizes, different elements, or element formulations are considered. This approach comparison is based on several selected model cases.

## 2. Experimental Testing Program

The flexural stability tests consisted of 47 concentrically loaded pin-ended specimens with a wide range of local and global slenderness. Sample lengths were chosen to provide a range of global slenderness values λ.

A detailed description of the comprehensive experimental program is well documented and described in the study of Mr. Buchanan et al. [38] pp. 298–303.

## 3. Numerical Finite Element Model

A parametrical numerical finite element model has been created in the ANSYS Classic technology (v 19.0.) [39] using APDL macros. The variable input parameters were all the material parameters (Table 1 and Table 2), geometrical parameters (Table 3, Table 4, Table 5, Table 6 and Table 7), as well as the initial global imperfection amplitude (*ω*_0_ + *e*_0_).

Two sets of material property values, referred to as SCP and TP, have been considered in this study. Both sets are based on the averaged values from all of the relevant available data: for the stub-column material properties (SCP, Table 1, based on the data from Table 4 in [38]) and for the tensile coupon material properties (TP, Table 2, based on the data from the Table 2 in [38]), respectively. *E*_0_ is the material elastic Young’s modulus, *σ*_0.2_ is the material 0.2% proof stress, and *n* is a strain-hardening exponent, *σ*_1.0_ is the 1% proof stress of the material, *n’*_0.2,1.0_ is a strain-hardening exponent, and *σ*_u_ is the ultimate tensile stress. Stress-strain relations defined by these parameters are elaborately described in Section 3.3.

Geometrical parameters, *D* (cross-section outer diameter), *L* (effective structural length, with the inclusion of the additional knife edge lengths), *t* (wall thickness), and the imperfection amplitudes (*ω*_0_ + *e*_0_) have been considered in accordance with the measured values of the respective specimens provided (Table 3, Table 4, Table 5, Table 6 and Table 7 below, based on the data from the Table 5, Table 6, Table 7, Table 8 and Table 9 in [38], respectively).

### 3.1. Geometry of the Numerical Models, Formulations of the Adopted Finite Elements

Several approach variants of the numerical model have been considered:

- #A = shell elements model, default mesh size, global imperfection only;
- #B = #A, however finer mesh;
- #C = #A + local imperfection;
- #D = solid elements model, default mesh size, three elements through the cross-section thickness *t*, selective reduced integration element formulation [39];
- #E = #D, however with “uniform reduced integration” [39] element formulation;
- #F = #D, however with “enhanced strain” [39] element formulation.

All the 74 finite element analyses (which differ in material and geometrical parameters) to validate the numerical model results have been conducted only considering only the modeling approach #A. The other model variants (#B–#F) have been conducted only for chosen geometries, and the results are documented and compared as well.

#### 3.1.1. Modeling Approach #A

**Figure 1:**
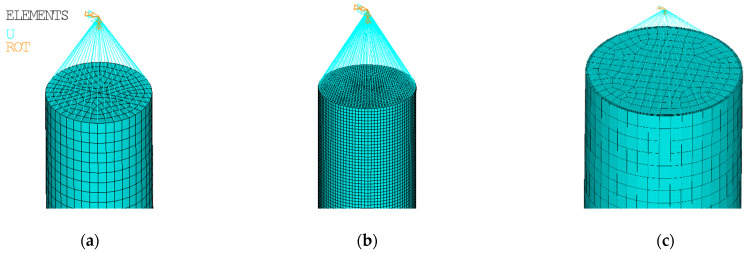
Mesh geometry examples for various modeling approaches: (**a**) #A, shell model, default mesh (88.9 × 2.6-400-P) [38]; (**b**) #B shell model, finer mesh (88.9 × 2.6-400-P) [38]; and (**c**) #D, solid model, default mesh (104 × 2-3080-P) [38].

Four-node structural shell elements (SHELL 181) with 3 translational degrees of freedom (DOF) and 3 rotational DOF per node have been used to model the CHS columns. The elements possess bending and membrane stiffness (Mindlin-Reissner theory). A reduced integration with hourglass control has been considered with one integration point (three through the thickness). The elements include the linear effect of transverse shear deformation. An assumed shear strain formulation of Bathe-Dvorkin is used to alleviate shear locking [39]. The geometrical shapes of all the shell elements used to model the CHS column are rectangles, with a maximal edge size of 8 mm in the longitudinal direction and 5 mm in the tangential direction (along the circumference) (Figure 1a). This differs from the mesh size adopted by Buchanan [38], where the longitudinal and circumferential dimensions of the mesh are considered the same as the wall thickness of the CHS tube, *t*.

#### 3.1.2. Modeling Approach #B

In this study, only a few selected cases have been reanalyzed considering a finer mesh (3 mm × 3 mm), and the results are discussed. The difference in mesh sizes is depicted in Figure 1a,b (depicted on the geometry of the specimen 88.9 × 2.6-400-P [38]).

#### 3.1.3. Modeling Approach #C

For these cases, initial local geometrical imperfections have also been considered (Section 3.2). The global imperfections and the mesh size are the same as in approach #A.

#### 3.1.4. Modeling Approach #D

Several model cases have been recalculated by adopting the solid structural finite elements (SOLID 185) for the CHS tube instead of the shell elements (Figure 1c). The mesh sizes in the longitudinal and tangential directions are the same as in approach #A. Three elements have been considered in the radial direction, along the cross-section thickness *t*. The default key option (0) of the element technology has been considered; therefore, there is a full integration with the B-bar method [39] (selective reduced integration) which helps to prevent volumetric locking in nearly incompressible cases.

#### 3.1.5. Modeling Approach #E

For few cases, the uniform reduced integration of the solid finite elements with hourglass control has been adopted. One integration point per solid element is considered in this formulation. Note: key option No. 2 is set up as 1 [39].

#### 3.1.6. Modeling Approach #F

To compare the results, the enhanced strain formulation of solid elements has been adopted for selected cases. Shear locking, as well as volumetric locking in nearly incompressible cases, are both prevented if this formulation is considered [39]. Certain numbers of internal degrees of freedom are introduced, and therefore, this option is less efficient. Note: key option No. 2 is equal to 2 [39].

### 3.2. Geometrical Imperfections

**Figure 2:**
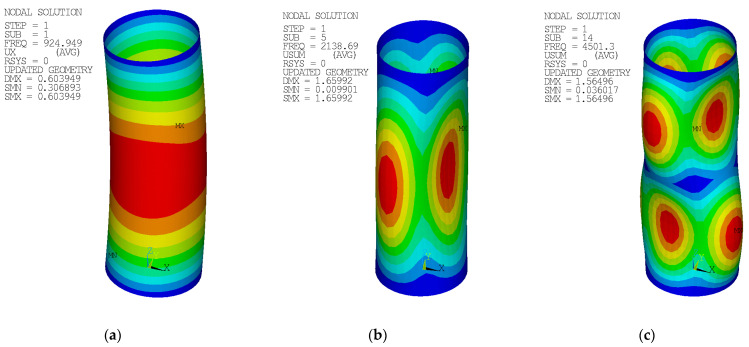
Example of the modal analysis results (geometry of 88.9 × 2.6-400-P) [38]: (**a**) Modal shape (displacements in the x-axis direction) for global imperfection; (**b**,**c**) Modal shapes (total displacement) to determine the local imperfection.

A global imperfection was incorporated into the FE model using the form of the lowest global buckling modal shape obtained from prior modal analysis (an example is given in Figure 2a). A global imperfection amplitude was used to simulate an initial global imperfection, as well as eccentricity. The amplitudes of *ω*_0_ + *e*_0_ (Table 3, Table 4, Table 5, Table 6 and Table 7) were considered. Local imperfections were generally neglected. Only in a few selected cases (#C), a local imperfection amplitude of *t*/10 was considered (where *t* is the section thickness). The local imperfection took a shape of the lowest local buckling modal shape (an example of 88.9 × 2.6-400-P [38] is in Figure 2b,c. Note: shape according to “b” was considered).

### 3.3. Material Model

To describe the behavior of the stainless-steel material, a stress-strain relation proposed by Ramberg and Osgood [10], modified by Hill [11] has been adopted:

(1) $$\varepsilon =\frac{\sigma }{E_{0}}+0.002\cdot {\left(\frac{\sigma }{\sigma_{0.2}}\right)}^{n},$$

where *σ* and *ε* are engineering stress and strain, respectively, *E*_0_ is the material elastic Young’s modulus, *σ*_0.2_ is the material 0.2% proof stress, and *n* is a strain-hardening exponent. At strains higher than *σ*_0.2_ value, this model overestimates the stress values [41]. A two-stage compound stress–strain curve devised by Mirambell and Real [42] provides a better agreement with experimental data for stress values above the 0.2% proof stress [41]. For the case of the compressive loading, a certain modification of the second stage is proposed by Gardner [33]:

(2) $$\varepsilon =\frac{\sigma -\sigma_{0.2}}{E_{0.2}}+\left(0.008-\frac{\sigma_{1.0}-\sigma_{0.2}}{E_{0.2}}\right)\cdot {\left(\frac{\sigma -\sigma_{0.2}}{\sigma_{1.0}-\sigma_{0.2}}\right)}^{n_{0.2,1.0}^{\prime }}+\varepsilon_{t0.2}\Leftrightarrow \sigma >\sigma_{0.2},$$

where *σ*_1.0_ is the 1% proof stress of the material, *n’*_0.2,1.0_ is a strain-hardening exponent, and *E*_0.2_ is the stiffness (tangent modulus) at the 0.2% proof stress given as:

(3) $$E_{0.2}=\frac{E_{0}}{1+0.002\cdot n\cdot E_{0}/\sigma_{0.2}}.$$

**Figure 3:**
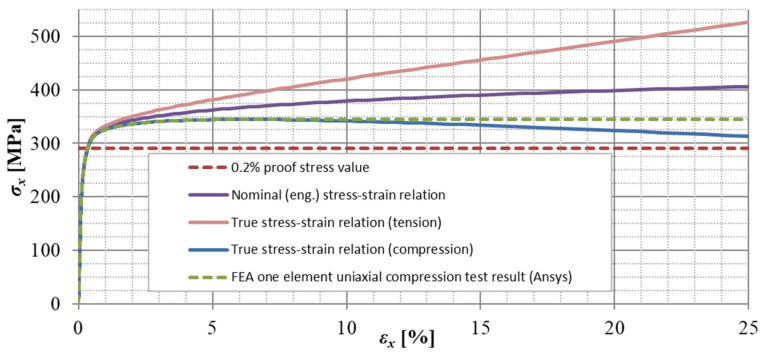
Material model verification by one element uniaxial tests (106 × 3-400-FR) [38].

For the finite element (FE) numerical analyses, a multilinear material model with isotropic hardening has been considered (Mises plasticity). A more detailed description is presented in the author’s previous study [14]. In this study, however, the stress-strain behavior only up to the value of 0.1 *σ*_0.2_ is considered as ideal elastic (to neglect plasticity at low strains), instead of the previously considered value of 2/3 *σ*_0.2_ [14] (which was too high for some cases). The engineering (nominal) stress-strain material curves have been transferred into true stress and logarithmic strain dependences to match the results of geometrically nonlinear FE analyses:

(4) $$\sigma_{true}=\sigma_{nom}\cdot (1+\varepsilon_{nom}),$$

(5) $$\varepsilon_{true}=\ln(1+\varepsilon_{nom}),$$

where *σ_nom_* is the nominal engineering stress, *ε_nom_* is the nominal engineering strain, and *ε_true_* is the true total (mechanical) strain. For the compressive material properties, *ε_nom_* have been introduced with negative values. As it is impossible to define a negative tangent of the stress-strain relation while adopting isotropic hardening [39], the stress-strain relation has been defined as ideal plastic (with its tangent slope close to 0 but positive) instead of any kind of softening after the peak stress. An example of material model verification (parameter values of 106 × 3-400-FR in Table 4 in [38]) is depicted in Figure 3.

In cold-formed CHS, small values of the membrane residual stresses have been observed, and as such can be neglected [43]. The residual stresses through thickness are implicitly incorporated by considering the measured values of the material properties [44].

### 3.4. Boundary Conditions and Loading

To simulate a pin-ended column, all the circumferential nodes at the end of the CHS tube are connected (in the radial direction) with a single node located on the section axis. The offset of the node is either 50 (40 + 10) or 77 mm in dependence on the experimental setups described in [38] p. 301. The nodal connections are modeled using rather stiff beam elements. Such a boundary condition is considered at both ends of the CHS tube (see Figure 1). All translational and 2 rotational degrees of freedom of the bottom node are constrained. The loading during the FEA has been conducted by a prescribed displacement of the upper node (in the direction of the CHS tube axis). Two translational and 2 rotational degrees of freedom of the upper node were constrained.

## 4. Results

The results of all the 74 FEA (finite element analyses) of the approach #A (shell elements), the ultimate axial loads *N_u,FE_*, and corresponding ultimate midheight lateral deflections *ω_u,FE_*, together with the experimental results (*N_u,exp_* and *ω_u,exp_*) [38], are documented in Table 8, Table 9, Table 10, Table 11 and Table 12.

**Figure 4:**
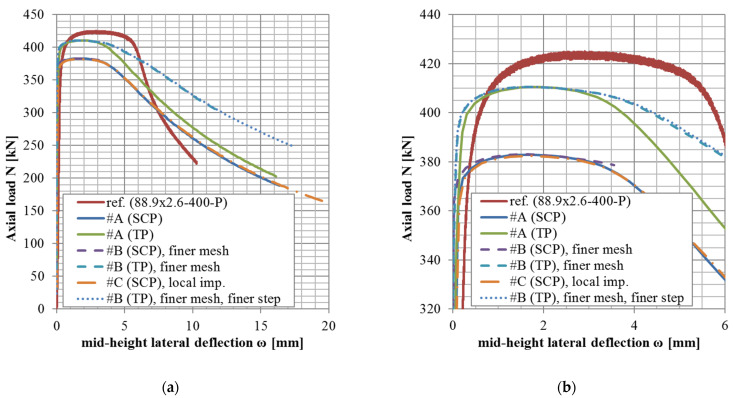
Experimental and FE loads vs. midheight lateral deflection curves of 88.9 × 2.6-400-P [38]: (**a**) whole*; (**b**) zoomed.

**Figure 5:**
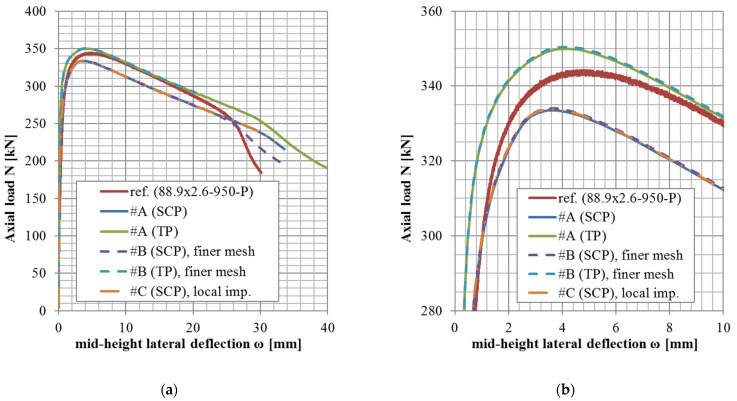
Experimental and FE loads vs. midheight lateral deflection curves of 88.9 × 2.6-950-P [38]: (**a**) whole; (**b**) zoomed.

**Figure 6:**
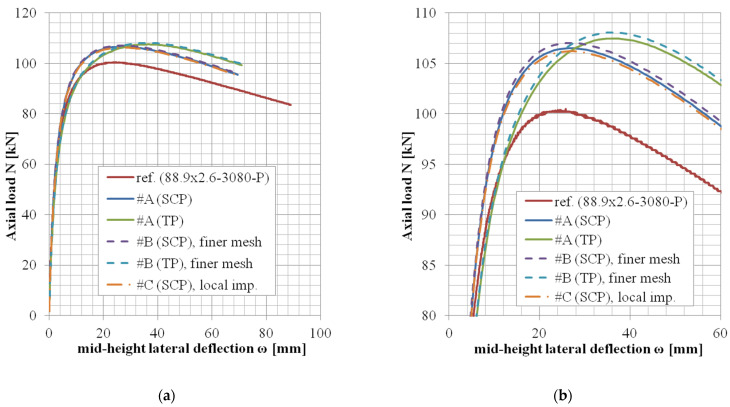
Experimental and FE loads vs. midheight lateral deflection curves of 88.9 × 2.6-3080-P [38]: (**a**) whole; (**b**) zoomed.

The results of variants in accordance with the approach #B or #C (shell elements, finer mesh variant, or local imperfection variant) are not documented in tables but rather only graphically (Figure 4, Figure 5 and Figure 6).

In the case of the modeling approach #D (solid elements, selective reduced integration), two whole sets of lengths (two chosen cross-sections) have been reanalyzed, and the results are summarized in Table 13 and Table 14. The difference between approaches #D and #A was very negligible in the matter of monitored data, and therefore, the remaining geometries have not been reanalyzed considering solid model (the presented results in Table 8 are almost the same as those in Table 13, and the results of Table 9 and Table 14 likewise). Statistical comparison of the monitored data (*N_u,FE_*/*N_u,exp_* and *ω_u,FE_*/*ω_u,exp_* values) between approaches #A and #D are summarized in Table 15. For approach #A, only the results which are presented in Table 8 and Table 9 are applicable for this statistical evaluation (as for approach #D, only the results of corresponding cross-section geometries are available—Table 13 and Table 14).

FE models of approach #A were validated by comparing the averaged normalized ultimate loads *N_u,FE_*/*N_u,exp_* and the averaged normalized deflections *ω_u,FE_*/*ω_u,exp_* (Table 16). The validation results are also compared with the results of Mr. Buchanan et al. [38].

Furthermore, the selected model cases of the different solid element formulations considered in approaches #E and #F have not seemed to yield significantly different results in the matter of the observed outputs and are therefore documented only graphically in this study.

Global slenderness $\bar{\lambda }$ is calculated in dependence on the compressive cross-section class (according to EN 1993-1-4 [15]) considering Equation (6) for section class (cl.) 1–3, and Equation (7) for cross-section class 4:

(6) $$\bar{\lambda }=\sqrt{\frac{A\cdot \sigma_{0.2}\cdot L^{2}}{\pi^{2}\cdot E\cdot I}},$$

(7) $$\bar{\lambda }=\sqrt{\frac{A_{eff}\cdot \sigma_{0.2}\cdot L^{2}}{\pi^{2}\cdot E\cdot I}},$$

where *A* is the cross-section area, *σ*_0.2_ is the 0.2% proof stress, *E* is Young’s modulus, *I* is the second moment of area, *L* is the effective length, and *A_eff_* is the effective cross-sectional area determined in accordance with the formula from BS 5950-1 [45]:

(8) $$A_{eff}=A\cdot {\left[\left(\frac{90}{D/t}\right)\cdot \left(\frac{235}{\sigma_{0.2}}\cdot \frac{E}{210000}\right)\right]}^{0.5}.$$

Compressive class slenderness limits have been considered in accordance with EN 1993-1-4 [15]. The global slenderness $\bar{\lambda }$ and the compressive classes (cl.) have been calculated for all the model geometries for both sets of material properties (SCP and TP) and are documented in Table 8, Table 9, Table 10, Table 11, Table 12, Table 13 and Table 14 (values of $\bar{\lambda }$ in Table 13 and Table 14 are, of course, the same as in Table 8 and Table 9, respectively).

**Figure 7:**
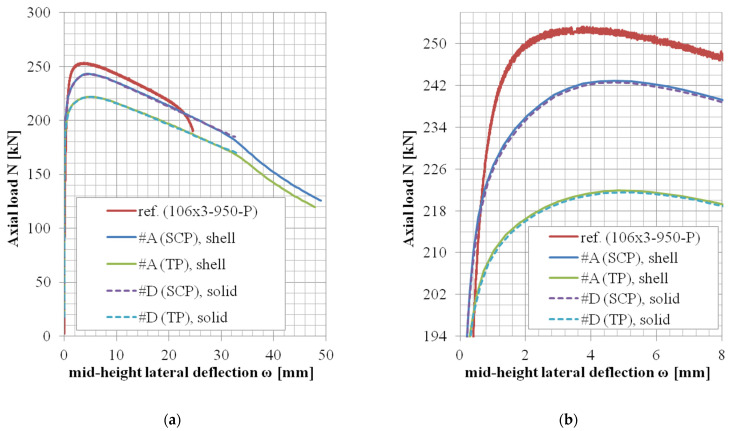
Experimental and FE loads vs. midheight lateral deflection curves of 106 × 3-950-P [38]: (**a**) whole; (**b**) zoomed.

**Figure 8:**
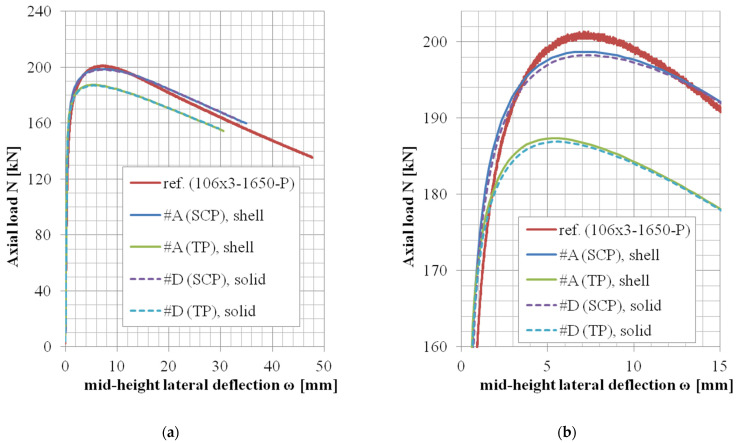
Experimental and FE loads vs. midheight lateral deflection curves of 106 × 3-1650-P [38]: (**a**) whole; (**b**) zoomed.

**Figure 9:**
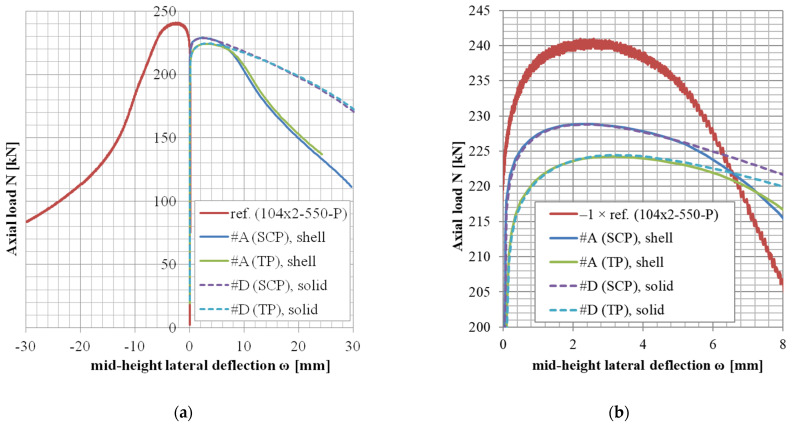
Experimental and FE load vs. midheight lateral deflection curves of 104 × 2-400-P [38]: (**a**) whole*; (**b**) zoomed.

**Figure 10:**
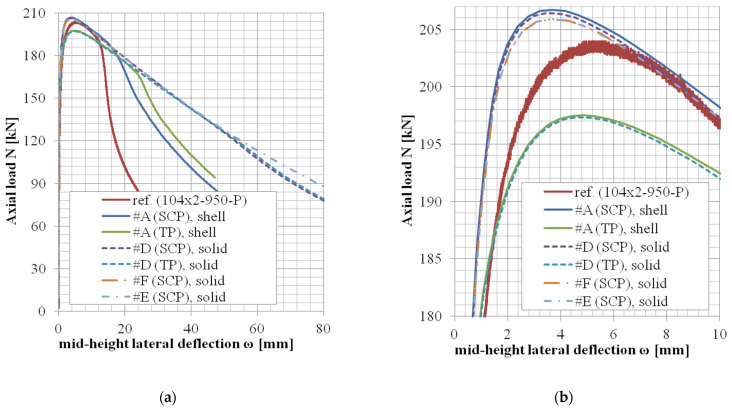
Experimental and FE load vs. midheight lateral deflection curves of 104 × 2-950-P [38]: (**a**) whole*; (**b**) zoomed.

**Figure 11:**
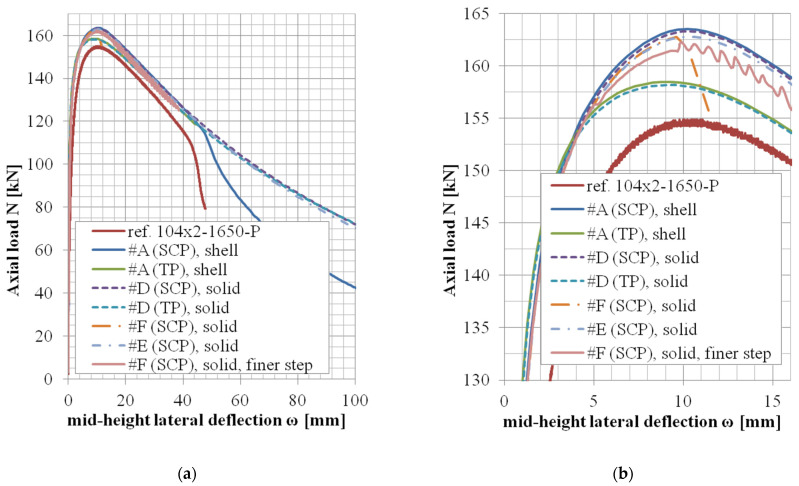
Experimental and FE loads vs. midheight lateral deflection curves of 104 × 2-1650-P [38]: (**a**) whole*; (**b**) zoom.

The *N*-*ω* (axial load—midheight deflection) curves are provided in Figure 4, Figure 5, Figure 6, Figure 7, Figure 8, Figure 9, Figure 10 and Figure 11. For each figure, the “a” part is used to depict the whole force-deflection curve, and the “b” (zoomed) part of each Figure focuses on the area near the peak forces *N_u,exp,_* and *N_u,FE_*, so the negligible differences of the *N*-*ω* curves in these areas between considered modeling approaches are also visible. In some cases, the “a” part of the figure is described as “whole*”-in these cases, certain *N*-*ω* curves of the graph are not depicted until the very last converged or analyzed step of that case.

The differences in the matter of the force-deflection curves between modeling approaches #A, #B, and #C (shell numerical model with default mesh size, finer mesh size, and local imperfection, respectively) are depicted for selected specimens of the cross-sectional set 88.9 × 2.6 in Figure 4, Figure 5 and Figure 6.

The differences in the matter of the force-deflection curves between modeling approaches #A and #D (shell vs. solid numerical model) are depicted for the selected specimens of the cross-sectional set 106 × 3 and 104 × 3 in Figure 7, Figure 8, Figure 9, Figure 10 and Figure 11. Moreover, in Figure 10 and Figure 11, the results of the approaches #E and #F (different solid element formulations) are also documented and available for comparison.

As described in [38], three specimens from those of shorter lengths (104 × 2-550-P, 104 × 2-750-P, and 106 × 3-550-P) changed the initial direction of the midheight lateral deflection (induced by the applied eccentricity) before reaching the peak force *N_u,exp_* (hence the minus values of *ω_u,exp_* in Table 8 and Table 9). One specimen (80 × 1.5-300-P) changed the deflection direction after the peak force *N_u,exp_* had been reached (hence positive value of the *ω_u,exp_* for this case in Table 11). Note: the deflection reference data of the specimen 104 × 2-550-P was multiplied by −1 only for the purpose of depiction in Figure 8b. Original data were considered for the statistical evaluation.

In the case of modeling approach #F (solid elements, enhanced strain formulation), the convergence of the numerical solution was more sensible to the step size of the analysis. When the step size in approach #F was adopted, the same as in all the other approaches, the last converged substeps were at the values of midheight lateral deflection *ω* approx. Six mm and 11.3 mm for specimens 104 × 2-950-P and 104 × 2-1650-P, respectively, (orange dash-dotted curves in Figure 10 and Figure 11; note: the last point of the orange curve in Figure 11 is also a converged state). To obtain further analysis data, a finer step size has been adopted for the case 104 × 2-1650-P, and the results are described by the pink curve in Figure 11. However, in the postpeak region of this *N*-*ω* curve, certain oscillations have developed. The amplitudes of these oscillations are not significantly higher than the oscillation amplitudes of the reference data (red curve). Further analyses of the approach #F have not been conducted due to much lower computational efficiency, as well as not a very significant difference in the matter of the monitored results (ultimate axial load *N_u,FE,_* and the corresponding midheight lateral deflection *ω_u,FE_*).

The differences in the results for approach #E are rather negligible near the areas of the ultimate axial loads *N_u,FE_* and noticeable only in the areas of large deformations. Therefore, further analyses considering this approach have not been conducted, either.

**Figure 12:**
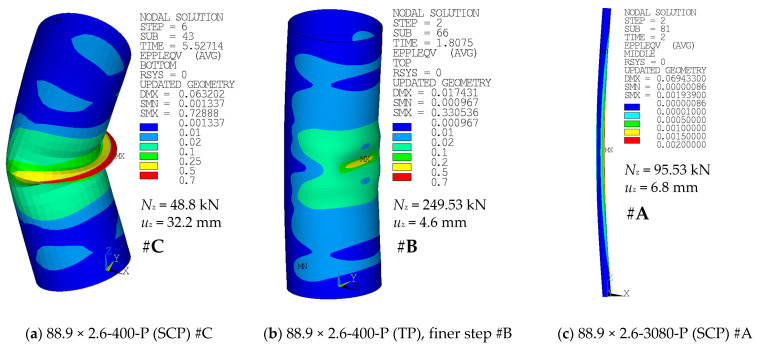
Equivalent plastic strain of various cases: (**a**) #C (SCP) curve from Figure 4; (**b**) #B (TP), finer step curve from Figure 4; and (**c**) #A (SCP) curve from Figure 6.

**Figure 13:**
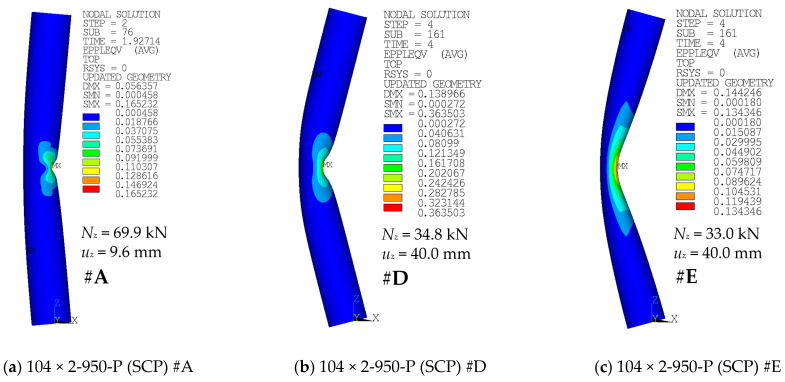
Equivalent plastic strains of various cases: (**a**) #A (SCP) curve from Figure 10; (**b**) #D (SCP) curve from Figure 10; and (**c**) #E (SCP) curve from Figure 10.

**Figure 14:**
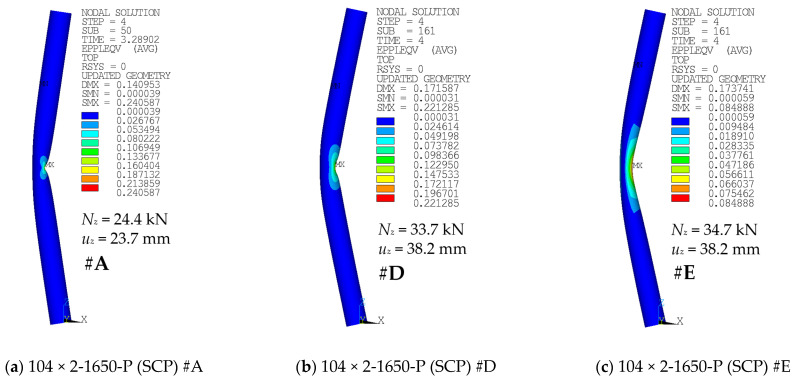
Equivalent plastic strains of various cases: (**a**) #A (SCP) curve from Figure 11; (**b**) #D (SCP) curve from Figure 11; and (**c**) #E (SCP) curve from Figure 11.

Contour plots of the equivalent plastic strains and, more importantly, deformed geometries for selected cases are depicted in Figure 12, Figure 13 and Figure 14. For each contour plot, the values of deformation load *u_z_* and the corresponding axial load *N_z_* are provided. Furthermore, the modeling approach is stated, and the link of the case with the corresponding F-u curve and figure number of the graph is provided.

Global buckling was the most common failure mode in the case of longer specimens (e.g., Figure 12c). Shorter specimens have rather developed a local buckle near the midheight of the compressed side of the tubular cross-section after the peak load has been reached (e.g., Figure 12a,b). The buckling modes are very similar to those described in the study by Buchanan et al. [38].

Figure 13 and Figure 14 depict the differences in the deformed shape at the compressed face near the midheight of the selected specimens when adopting the modeling approach of #A (shell elements), #D (solid elements), or #E (solid elements with uniform reduced integration). The area near the local buckling is deformed differently in these cases.

**Figure 15:**
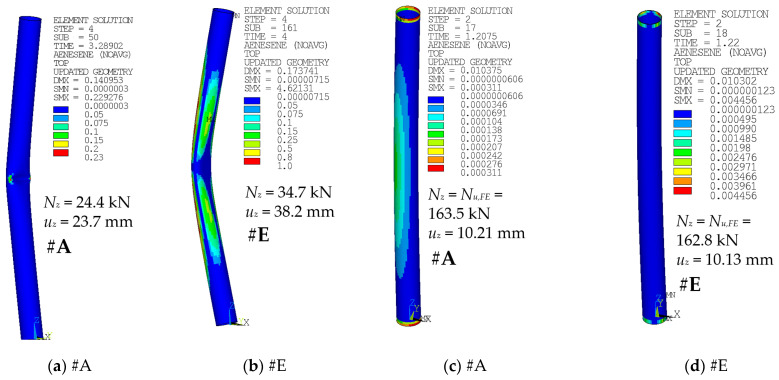
104 × 2-1650-P (SCP), ratio of artificial to total energy: (**a**) #A, last step; (**b**) #E, last step; (**c**) #A, at peak load; and (**d**) #E, at peak load.

Figure 15 depicts energy ratios of the case 104 × 2-1650-P (SCP) modeled by approaches #A and #E. The energy ratios are monitored at states of the last analyzed steps (Figure 15a,b), and also at the states of the maximal peak axial loads *N_u_* (Figure 15c,d). In the course of the computation, the artificial energy is introduced to sustain hourglass control of the reduced integration elements, either shells from case #A or solids from case #E. In the case of modeling approaches #D (selective reduced integration) or #F (full integration), no artificial energy is being introduced. The solution is generally acceptable if the ratio of artificial energy to the total energy is less than 5% [39]. Mesh refinement is recommended otherwise.

Figure 15 depicts the ratio of the artificial to total energy for each element. In the case of approach #A, the recommended value of 5% has been exceeded in the last step of the analysis only locally, with the maximal value of 23% in two shell elements (Figure 15a). However, in the modeling approach #E, the limit value has been exceeded in a significant number of the finite elements (Figure 15b). In the step where the peak loads *N_u_* have been achieved (Figure 15c for approach #A and Figure 15d for the approach #E), the energy ratios are far below the recommended value of 5%, with generally lower values for the modeling approach case #A (shell elements). Note: pay attention to the different contour scales.

## 5. Discussion

### 5.1. Validation Data (Approach #A)

The average and COV (coefficient of variation) values of normalized ultimate load *N_u,FE_*/*N_u,exp_* and deflection *ω_u,FE_*/*ω_u,exp_* are depicted in Table 16 (based on the values from Table 8, Table 9, Table 10, Table 11 and Table 12). These results are in good agreement with the research of Mr. Buchanan et al. [38] (also depicted in Table 16). The mean value of the normalized ultimate load *N_u,FE_*/*N_u,exp_* is close to one for both sets of the material properties, TP and SCP (tensile properties, stub-column properties), and the COV values are close to zero; therefore, it is possible to consider the accuracy as sufficient. COV is smaller when the material set of SCPs has been adopted in the FEA, the same as observed in the study by Buchanan et al. [38]. The value of the normalized ultimate deflection *ω_u,FE_*/*ω_u,exp_* is closer to one when the SCP set of material properties has been considered, again in accordance with the conclusions in [38]. The values of *ω_u,FE_*/*ω_u,exp_* are 0.627 and 0.660 for TP and SCP, respectively, and are in accordance with the result values of 0.612 and 0.708 from [38]. These values seem to be rather far from the accurate match (the value of 1), with quite a high coefficient of variation: 1.006–0.825 in this study and 0.920–0.834 in the study [38]. This is firstly due to the flat shape of the load-deflection curves near their maximum. Secondly and more importantly, as a consequence of the lateral deflection in the direction opposite to the initial geometrical imperfection before reaching the peak value of the axial force, which was observed in three (out of 37) reference cases, hence considered with the negative value (also see [38]). Note: in the fourth reference case, the deflection evolved into the opposite direction after reaching the peak load, and therefore, is not considered with the negative value of the lateral deflection [38].

**Figure 16:**
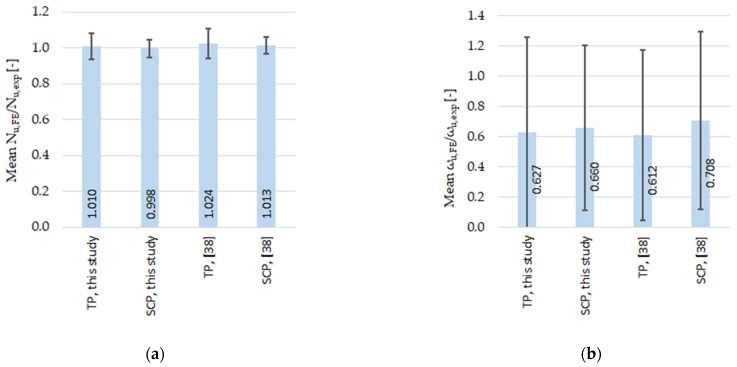
Average values with standard deviation bars for: (**a**) normalized ultimate loads; (**b**) normalized ultimate deflections; both depicted for the results considering SCP and TP material properties, based on this study and also the study by Mr. Buchanan [38].

Statistical values, the average (mean) value of the ultimate load *N_u,FE_*/*N_u,exp_*, and deflection *ω_u,FE_*/*ω_u,exp_* along with the standard deviations bars are graphically depicted in Figure 16 (based on Table 16). These values are based on the results of this study and of the study by Mr. Buchanan [38].

In general, the validation data are very similar to the values discussed in [38], and therefore, the considered numerical FE model is possible to be considered as validated with no dispute. Consistency between FEA results and the experimental data has been achieved (especially for the case of SCP material properties depicted in Figure 8).

### 5.2. Mesh Size Influence (Approach #A vs. #B)

The difference between these two mesh sizes, coarser (8 mm × 5 mm, approach #A) or finer (3 mm × 3 mm approach #B), has been observed for the geometries of three different slenderness values from the set of the same cross-section (Figure 4, Figure 5 and Figure 6, cross-section 88.9 × 2.6), considering the lowest structural length from the set (400 mm, Figure 4), the largest one (3080 mm, Figure 6), and the third value from (950 mm, Figure 5). This choice of various lengths for one selected cross-section is being considered as a sufficiently representative sample (all the cross-sections of this study are geometrically very similar).

Only in the case of little slenderness, the initial model stiffness is considerably larger when the finer mesh has been adopted (Figure 4). This might be the reason for the adopted boundary conditions as well (the connection of the constrained nodes with the circumferential nodes at the CHS tube ends), with possibly larger influence in cases of the shorter specimens. The influence is greater for finer meshes due to the utilized automatized APDL macro (finer mesh = more circumferential nodes = more beams to connect the circumferences with the appropriate loading nodes). This issue is not considered a concern, as the real stiffness of the boundary conditions was not determined with certainty. In addition, the postpeak behavior is slightly different, when the finer mesh has been considered only in a few cases (Figure 5). In some model cases, the analyses considering the finer mesh had more convergence difficulties while the same time step was considered (e.g., Figure 4, #B, SCP).

Based on the results of these selected geometries, the difference between various mesh sizes is very negligible in the matter of the monitored results (*N_u_*, *ω_u_*), which are the principal interest of this study.

### 5.3. Local Imperfection Influence (Approach #A vs. #C)

Influences of the local initial imperfections are investigated for the same selected cases as described previously (Section 5.2). The difference between cases with adopted and neglected local imperfection is very small (Figure 4, Figure 5 and Figure 6) for all the cases. Therefore, the local imperfections have not been implemented for the other cases to conduct the general validation of the numerical FE model. The effort invested in the implementation of the local initial geometrical imperfections is not meaningful for the objectives of this study. For different lengths of the column specimens, different modal shapes might be applicable to incorporate the local imperfection. Therefore, a manual check of the modal shapes themselves is required. On the other hand, the global imperfection shape has always been obtained from the first modal shape, which is much more feasible if the analysis process is to be automatized (e.g., via APDL macros).

### 5.4. Shell or Solid FE Model (Approach #A vs. #D)

The comparison between the shell model and the solid model in the matter of the globally monitored results (normalized ultimate load *N_u,FE_*/*N_u,exp_* and deflection *ω_u,FE_*/*ω_u,exp_*) are depicted in Table 15. In the case of approach #D (solid model), two whole sets of lengths (considering two different cross-sections) have been reanalyzed. These results are summarized in Table 13 and Table 14 (and are very similar to the corresponding results of case #A, Table 8 and Table 9, respectively). The mean values and the values of the coefficient of variations are practically the same for both approaches (Table 15). Load-deflection curves are compared in Figure 7, Figure 8, Figure 9, Figure 10 and Figure 11. The behavior in the postpeak softening region (where the large strains are involved) is different for some modeled cases. However, this is not the objective of this study.

### 5.5. Full Integration of the Solid Elements (approach #F)

This approach has been investigated for only two selected cases, the cross-section of 104 × 2, and structural lengths of 950 mm (Figure 10) and 1650 mm (Figure 11). When the same step size (and the same maximal number of Newton-Rhapson iterations within one step) has been considered, the convergence problems occurred shortly after reaching the peak force *N_u,FE_*. A finer time step needed to be implemented to achieve the convergence after the peak load. This has been introduced for one case (Figure 11). Although the convergence has been secured, certain oscillations have developed. The amplitude of these oscillations is not significantly higher than the oscillation amplitudes of the reference data, and therefore, the results are considered sufficiently good. Further analyses considering this approach, however, have not been conducted due to much higher computational demands. Moreover, the investigated results (*N_u_*, *ω_u_*) are very similar to those obtained by much more efficient approaches.

### 5.6. Uniform Reduced Integration (Approach #E), Selective Reduced Integration (Approach #D), Shell Elements with Reduced Integration (Approach #A)

The differences in the monitored results are very negligible, again (Figure 10 and Figure 11). A noticeable difference is in the shape of the local buckling area in the midspan of the column height (Figure 13 and Figure 14). The distortion in this area seems to be rather smoother for cases where the #E approach has been considered (Figure 13c or Figure 14c), in comparison with the sharper area of local buckling when either the #D approach (Figure 13b or Figure 14b) or the default #A approach (Figure 13a or Figure 14a) have been adopted.

The energy ratio (artificial to total energy) [39] is depicted in Figure 15. In order to consider the results, where the elements with the reduced integration have been considered as good, this energy ratio needs to be below 0.05 (5%), as recommended in [39]. The ratio is below the recommended value at the time of the peak load for both cases, #A (Figure 15c) and #E (Figure 15d), with slightly better (lower) values for approach #A. For the last observed step, however, the energy ratio exceeds 0.05 in a large number of the elements when approach #E has been adopted (Figure 15b), and only in few elements in the case of approach #A (Figure 15a). For the modeling approach #D, there is no additional artificial energy (this approach is considered to be more precise). Therefore, the postpeak results of approach #A are considered more convenient.

### 5.7. Other Observations

In all of the modeled cases, the region with the largest plasticity has developed in the midspan, on the compressed face of the columns. Examples are depicted in Figure 12. The shape of this area was slightly different when the finer mesh has been adopted (Figure 12b), compared to the coarser mesh of the same geometry (Figure 12a). In addition, the difference in the shape of the high plasticity area is between cases of various structural lengths (Figure 12 and Figure 13). For the large lengths (Figure 12c), the global buckling occurred much sooner, and the analyses have not been conducted long enough to observe any local distortion of the cross-sectional shape.

Although proper statistical evaluation among all the discussed modeling approaches is not provided, further cases have not been reanalyzed, as the results of approach #A are very satisfactory, compared to the results documented in the study by Mr. Buchanan [38], which was the main objective of this study.

### 5.8. Further Application

In future work, the initial imperfections will be considered as random variables. This will make it possible to study their influence on the ultimate limit state using stochastic sensitivity analysis [46,47,48,49], where the main influences and interaction effects between imperfections can be the subject of research.

## 6. Conclusions

The performed simulations aimed at the comparison of advanced numerical models and the determination of their limitations in the simulation of the pressure test of thin-walled stainless-steel columns, considering the loss of stability. The study shows a significant effect of the correct description of boundary conditions on the overall response. In addition to the comparative (normalized ultimate load *N_u,FE_*/*N_u,exp_* and deflection *ω_u,FE_*/*ω_u,exp_*), several generalizable conclusions could be obtained by cross-comparison, which is discussed in detail in Section 5. Experimentally determined data from extensive research were considered as reference values. Very good agreement was reached for all used models.

The mean (average) value of the normalized ultimate axial load *N_u,FE_*/*N_u,exp_* was 1.010 (standard deviation 0.074) in this study and 1.024 (standard deviation 0.083) in the study by Mr. Buchanan, for the numerical analyses based on the “tensile test material properties” (TP) values. In the case of numerical simulations based on the values of the “stub-column material properties” (SCP), the average normalized ultimate axial load was 0.998 (standard deviation 0.050) in this study and 1.013 (standard deviation 0.047) in the study by Mr. Buchanan et al. These statistical values are graphically depicted in Figure 16a (and also in Table 16). The values of normalized ultimate forces are very close to 1, and the standard deviation is also low. The validation results for the ultimate forces presented here are in good agreement with the experimental study.

In the case of the normalized ultimate deflection *ω_u,FE_*/*ω_u,exp_*, the average values for numerical analyses utilizing the TP material values are 0.627 (st. dev. 0.631) and 0.612 (st. dev. 0.563) for this study and the study conducted by Mr. Buchanan et al., respectively. For numerical simulations utilizing the SCP values of material properties, the average values are 0.660 (st. dev. 0.545) and 0.708 (st. dev. 0.591) for this study and the study of Mr. Buchanan et al., respectively. These values are graphically depicted in Figure 16b (and in Table 16). The values seem to be rather scattered (normalized average not close to 1.00, large deviation). However, these values are very similar to those from the study by Mr. Buchanan et al., where the finite element models are successfully validated. The large deviation of values is the reason for the flat nature of the load-deflection curve near its maximal value of the ultimate load; therefore, larger values of standard deviations are expected.

Another objective was to describe a robust design procedure with the appropriate use of numerical simulation. This was achieved by a comparison of several modeling approaches. The modeling approaches differ in the adopted type of finite elements (either shell or solid), in varying element formulations (e.g., full integration or reduced integration), in varying mesh sizes (finer, coarser) or local geometrical imperfection incorporation or absence. The effects of these modeling approaches on the monitored main objective model outputs (ultimate axial load *N_u_* and the corresponding midheight lateral deflection *ω_u_*) are relatively very small and therefore negligible. Considering the shape of the whole force-deflection curve and the local buckling area, the type of adopted finite elements along with their formulation set up have the highest importance on the numerical model response. The most suitable, and also the most effective, are shell elements. The influence of the mesh size (considering two types of mesh, either the finer 3 mm × 3 mm or coarser 5 mm × 8 mm) is slightly more important for shorter specimens than for the longer ones. The most negligible influence on the results, in general, is caused by the effect of the local geometrical imperfection within the considered amplitude value of *t*/10 (where *t* is the section thickness—various value for various cross-sectional sets).

The validated numerical FE models presented here will be used for future analyses, where a study of initial imperfections influence on the ultimate limit state using stochastic sensitivity analysis will be conducted. These are simulations of large sets of samples, and therefore, it is necessary to choose the most concise computational model with the minimum time requirement.

## Figures and Tables

**Table 1:** Summary of the stub-column material properties (SCP).

Cross-Sectional Set [38]	*E*_0_ (GPa)	*σ*_0.2_ (MPa)	*σ*_1.0_ (MPa)	*n* (-)	*n’*_0.2,1.0_ (-)	*σ*_u_ (MPa)
106 × 3 (SCP)	196.05	283.50	323.50	8.60	3.65	615.00
104 × 2 (SCP)	202.05	359.50	407.00	4.80	2.88	726.25
88.9 × 2.6 (SCP)	217.95	579.50	633.00	4.50	2.45	846.50
80 × 1.5 (SCP)	218.75	360.00	375.50	5.10	1.60	438.00
101.6 × 1.5 (SCP)	219.55	337.00	346.00	5.60	2.40	467.00

**Table 2:** Summary of the tensile coupon material properties (TP).

Cross-Sectional Set [38]	*E*_0_ (GPa)	*σ*_0.2_ (MPa)	*σ*_1.0_ (MPa)	*n* (-)	*n’*_0.2,1.0_ (-)	*σ*_u_ (MPa)
106 × 3 (TP)	210.10	261.00	297.50	11.50	3.65	615.00
104 × 2 (TP)	200.00	345.75	400.25	6.63	2.88	726.25
88.9 × 2.6 (TP)	197.20	624.50	681.00	8.85	2.45	846.50
80 × 1.5 (TP)	222.05	371.00	386.50	8.70	1.60	438.00
101.6 × 1.5 (TP)	224.30	358.00	375.50	14.40	2.40	467.00

**Table 3:** Measured and calculated geometric properties of the cross-section set 106 × 3 CHS (circular hollow section) pin-ended columns (austenitic steel).

Specimen [38]	*D* (mm)	*t* (mm)	*L* (mm)	*L/(ω*_0_ + *e*_0_) (-)	*ω*_0_ + *e*_0_ (mm)	*D/t* (-)	*A* (mm²)	*I* (mm⁴)
106 × 3-550-P	105.74	2.78	554.27	1218	0.455	38.04	899.2	1 192 413.2
106 × 3-750-P	105.81	2.88	754.21	1034	0.729	36.74	931.2	1 234 292.7
106 × 3-750-PR	105.77	2.72	754.21	880	0.857	38.89	880.5	1 169 702.0
106 × 3-950-P	105.78	2.79	954.00	957	0.997	37.91	902.7	1 197 754.6
106 × 3-1150-P	105.84	2.83	1154.00	1000	1.154	37.40	915.8	1 215 660.0
106 × 3-1650-P	105.63	2.74	1657.00	945	1.753	38.55	885.6	1 172 837.4
106 × 3-2150-P	105.88	2.71	2152.90	997	2.159	39.07	878.3	1 169 469.8
106 × 3-2650-P	105.64	2.73	2652.50	987	2.687	38.70	882.6	1 169 232.2
106 × 3-3080-P	105.67	2.70	3083.00	1044	2.953	39.14	873.4	1 158 388.6

**Table 4:** Measured and calculated geometric properties of the cross-section set 104 × 2 CHS pin-ended columns (austenitic steel).

Specimen [38]	*D* (mm)	*t* (mm)	*L* (mm)	*L/(ω*_0_ + *e*_0_) (-)	*ω*_0_ + *e*_0_ (mm)	*D/t* (-)	*A* (mm²)	*I* (mm⁴)
104 × 2-550-P	103.97	1.89	553.77	1180	0.469	55.01	606.1	789 755.3
104 × 2-750-P	104.01	1.89	753.84	887	0.850	55.03	606.3	790 683.8
104 × 2-950-P	103.97	1.88	954.00	827	1.154	55.30	602.9	785 804.7
104 × 2-1150-P	104.14	1.86	1153.50	1115	1.035	55.99	597.6	781 787.3
104 × 2-1650-P	104.09	1.85	1656.60	963	1.720	56.26	594.2	776 669.6
104×2-2150-P	104.08	1.79	2153.60	999	2.156	58.15	575.2	752 567.4
104 × 2-2650-P	103.92	1.75	2653.50	1064	2.494	59.38	561.7	733 154.5
104 × 2-3080-P	104.10	1.79	3084.00	1036	2.977	58.16	575.3	753 008.9

**Table 5:** Measured and calculated geometric properties of the cross-section set 88.9 × 2.6 CHS pin-ended columns (duplex steel).

Specimen [38]	*D* (mm)	*t* (mm)	*L* (mm)	*L/(ω*_0_ + *e*_0_) (-)	*ω*_0_ + *e*_0_ (mm)	*D/t* (-)	*A* (mm²)	*I* (mm⁴)
88.9 × 2.6-400-P	88.63	2.37	403.90	824	0.490	37.40	642.2	597 811.6
88.9 × 2.6-550-P	88.63	2.35	553.83	1309	0.423	37.71	636.9	593 171.4
88.9 × 2.6-750-P	88.78	2.41	753.93	944	0.799	36.84	653.9	610 244.2
88.9 × 2.6-950-P	88.77	2.37	954.00	1075	0.887	37.46	643.3	600 725.6
88.9 × 2.6-1150-P	88.77	2.37	1154.00	1142	1.011	37.46	643.3	600 725.6
88.9 × 2.6-1650-P	88.63	2.35	1656.60	1022	1.621	37.71	636.9	593 171.4
88.9 × 2.6-2150-P	88.77	2.30	2152.80	716	3.007	38.60	624.8	584 374.5
88.9 × 2.6-2650-P	88.72	2.33	2653.40	993	2.672	38.08	632.3	590 366.9
88.9 × 2.6-3080-P	88.67	2.32	3082.50	1027	3.001	38.22	629.3	587 013.8

**Table 6:** Measured and calculated geometric properties of the cross-section set 80 × 1.5 CHS pin-ended columns (ferritic steel).

Specimen [38]	*D* (mm)	*t* (mm)	*L* (mm)	*L/(ω*_0_ + *e*_0_) (-)	*ω*_0_ + *e*_0_ (mm)	*D/t* (-)	*A* (mm²)	*I* (mm⁴)
80 × 1.5-300-P	80.00	1.34	299.4	1270	0.236	59.70	331.1	256 184.3
80 × 1.5-450-P	80.01	1.34	448.9	1290	0.348	59.71	331.1	256 282.0
80 × 1.5-700-P	80.04	1.34	698.6	1151	0.607	59.73	331.3	256 575.3
80 × 1.5-900-P	80.03	1.34	899.1	1068	0.842	59.72	331.2	256 477.5
80 × 1.5-1100-P	80.13	1.34	1099.1	1109	0.991	59.80	331.6	257 456.3
80 × 1.5-1600-P	80.00	1.34	1599.3	1019	1.569	59.70	331.1	256 184.3

**Table 7:** Measured and calculated geometric properties of the cross-section set 101.6 × 1.5 CHS pin-ended columns (ferritic steel).

Specimen [38]	*D* (mm)	*t* (mm)	*L* (mm)	*L/(ω*_0_ + *e*_0_) (-)	*ω*_0_ + *e*_0_ (mm)	*D/t* (-)	*A* (mm²)	*I* (mm⁴)
101.6 × 1.5-350-P	101.66	1.34	349.4	917	0.381	75.87	422.3	531 379.4
101.6 × 1.5-500-P	101.68	1.34	499.6	1183	0.422	75.88	422.4	531 697.3
101.6 × 1.5-800-P	101.70	1.34	799.3	1180	0.677	75.90	422.4	532 015.2
101.6 × 1.5-1100-P	101.82	1.34	1099.5	1079	1.019	75.99	422.9	533 925.7
101.6 × 1.5-1600-P	101.71	1.34	1599.3	1124	1.423	75.90	422.5	532 174.3

**Table 8:** Approach #A, FEA, and experiment results of the cross-section set 106 × 3 CHS pin-ended columns.

Specimen [38]	Material Set SCP				Material Set TP				Experiment	
	$\bar{\lambda }$	cl.	*N_u,FE1_* (kN)	*ω_u,FE1_* (mm)	$\bar{\lambda }$	cl.	*N_u,FE2_* (kN)	*ω_u,FE2_* (mm)	*N_u,exp_* (kN)	*ω_u,exp_* (mm)
106 × 3-550-P	0.18	1	270.6	2.61	0.17	1	247.9	2.92	267.0	−9.56
106 × 3-750-P	0.25	1	265.0	3.33	0.23	1	241.4	4.20	244.8	4.02
106 × 3-750-PR	0.25	2	249.2	3.38	0.23	1	227.2	4.27	242.2	4.66
106 × 3-950-P	0.32	1	242.9	4.74	0.29	1	221.8	4.83	253.4	3.76
106 × 3-1150-P	0.38	1	234.1	6.13	0.36	1	214.9	5.67	248.8	4.10
106 × 3-1650-P	0.55	1	198.7	7.35	0.51	1	187.3	5.25	201.3	7.10
106 × 3-2150-P	0.71	2	176.2	7.97	0.66	1	170.4	5.88	185.5	10.66
106 × 3-2650-P	0.88	2	156.6	9.84	0.82	1	155.2	7.07	159.4	13.27
106 × 3-3080-P	1.02	2	139.0	11.70	0.95	1	140.4	9.31	150.8	10.57

**Table 9:** Approach #A, FEA, and experiment results of the cross-section set 104 × 2 CHS pin-ended columns.

Specimen [38]	Material Set SCP				Material Set TP				Experiment	
	$\bar{\lambda }$	cl.	*N_u,FE1_* (kN)	*ω_u,FE1_* (mm)	$\bar{\lambda }$	cl.	*N_u,FE2_* (kN)	*ω_u,FE2_* (mm)	*N_u,exp_* (kN)	*ω_u,exp_* (mm)
104 × 2-550-P	0.21	3	228.8	2.24	0.20	3	224.2	3.12	241.1	−2.21
104 × 2-750-P	0.28	3	218.9	2.44	0.28	3	211.1	3.63	232.1	−1.95
104 × 2-950-P	0.35	3	206.7	3.68	0.35	3	197.5	4.84	204.2	5.17
104 × 2-1150-P	0.43	3	195.9	5.06	0.42	3	186.7	5.54	180.8	6.24
104 × 2-1650-P	0.62	3	163.5	10.21	0.61	3	158.4	9.30	154.8	10.50
104 × 2-2150-P	0.79	4	132.3	14.05	0.79	3	132.4	9.79	126.4	15.19
104 × 2-2650-P	0.97	4	108.5	15.35	0.97	4	111.5	11.59	109.0	19.40
104 × 2-3080-P	1.14	4	95.4	17.84	1.13	3	99.2	14.10	89.7	22.81

**Table 10:** Approach #A, FEA, and experiment results of the cross-section set 88.9 × 2.6 CHS pin-ended columns.

Specimen [38]	Material Set SCP				Material Set TP				Experiment	
	$\bar{\lambda }$	cl.	*N_u,FE1_* (kN)	*ω_u,FE1_* (mm)	$\bar{\lambda }$	cl.	*N_u,FE2_* (kN)	*ω_u,FE2_* (mm)	*N_u,exp_* (kN)	*ω_u,exp_* (mm)
88.9 × 2.6-400-P	0.22	3	382.8	1.59	0.23	4	410.4	1.89	425.2	2.91
88.9 × 2.6-550-P	0.30	3	370.9	1.25	0.31	4	394.6	1.55	404.6	2.60
88.9 × 2.6-750-P	0.41	3	361.6	2.08	0.43	4	378.7	2.83	389.6	2.75
88.9 × 2.6-950-P	0.51	3	333.4	3.82	0.54	4	349.9	4.20	344.4	4.54
88.9 × 2.6-1150-P	0.62	3	306.9	5.88	0.65	4	325.3	5.65	295.3	8.09
88.9 × 2.6-1650-P	0.89	3	232.4	12.03	0.93	4	257.6	7.59	243.4	10.32
88.9 × 2.6-2150-P	1.15	4	168.9	17.60	1.20	4	184.2	15.25	164.7	19.79
88.9 × 2.6-2650-P	1.42	4	134.0	20.95	1.49	4	140.1	24.23	126.4	20.63
88.9 × 2.6-3080-P	1.65	4	106.5	26.39	1.73	4	107.4	36.61	100.5	25.81

**Table 11:** Approach #A, FEA, and experiment results of the cross-section set 80 × 1.5 CHS pin-ended columns.

Specimen [38]	Material Set SCP				Material Set TP				Experiment	
	$\bar{\lambda }$	cl.	*N_u,FE1_* (kN)	*ω_u,FE1_* (mm)	$\bar{\lambda }$	cl.	*N_u,FE2_* (kN)	*ω_u,FE2_* (mm)	*N_u,exp_* (kN)	*ω_u,exp_* (mm)
80 × 1.5-300-P	0.14	3	121.3	0.09	0.14	3	124.8	0.09	126.1	0.17
80 × 1.5-450-P	0.21	3	117.5	0.49	0.21	3	120.6	0.72	119.4	1.71
80 × 1.5-700-P	0.32	3	112.7	1.29	0.33	3	114.3	1.69	111.1	2.89
80 × 1.5-900-P	0.42	3	105.7	2.94	0.42	3	108.1	3.35	105.8	3.36
80 × 1.5-1100-P	0.51	3	98.9	4.68	0.51	3	102.3	4.32	97.8	4.52
80 × 1.5-1600-P	0.74	3	79.6	9.89	0.75	3	86.6	6.19	77.9	8.76

**Table 12:** Approach #A, FEA, and experiment results of the cross-section set 101.6 × 1.5 CHS pin-ended columns.

Specimen [38]	Material Set SCP				Material Set TP				Experiment	
	$\bar{\lambda }$	cl.	*N_u,FE1_* (kN)	*ω_u,FE1_* (mm)	$\bar{\lambda }$	cl.	*N_u,FE2_* (kN)	*ω_u,FE2_* (mm)	*N_u,exp_* (kN)	*ω_u,exp_* (mm)
101.6 × 1.5-350-P	0.12	4	141.0	0.07	0.12	4	151.7	0.09	148.6	1.07
101.6 × 1.5-500-P	0.17	4	140.7	0.44	0.17	4	149.7	1.26	145.4	2.34
101.6 × 1.5-800-P	0.27	4	136.5	1.10	0.27	4	142.7	2.43	137.5	2.70
101.6 × 1.5-1100-P	0.37	4	127.8	3.51	0.38	4	135.4	3.26	121.2	4.92
101.6 × 1.5-1600-P	0.54	4	111.9	7.73	0.55	4	124.0	4.33	104.1	8.39

**Table 13:** Approach #D, FEA and experiment results of the cross-section set 106 × 3 CHS pin-ended columns.

Specimen [38]	Material Set SCP				Material Set TP				Experiment	
	$\bar{\lambda }$	cl.	*N_u,FE1_* (kN)	*ω_u,FE1_* (mm)	$\bar{\lambda }$	cl.	*N_u,FE2_* (kN)	*ω_u,FE2_* (mm)	*N_u,exp_* (kN)	*ω_u,exp_* (mm)
106 × 3-550-P	0.18	1	270.2	2.40	0.17	1	247.7	2.96	267.0	−9.56
106 × 3-750-P	0.25	1	264.6	3.53	0.23	1	241.1	4.07	244.8	4.02
106 × 3-750-PR	0.25	2	248.8	3.58	0.23	1	226.9	4.14	242.2	4.66
106 × 3-950-P	0.32	1	242.5	4.70	0.29	1	221.5	4.86	253.4	3.76
106 × 3-1150-P	0.38	1	233.6	6.22	0.36	1	214.4	5.84	248.8	4.10
106 × 3-1650-P	0.55	1	198.2	7.35	0.51	1	186.9	5.66	201.3	7.10
106 × 3-2150-P	0.71	2	175.7	8.19	0.66	1	169.9	6.12	185.5	10.66
106 × 3-2650-P	0.88	2	156.1	9.25	0.82	1	154.6	7.36	159.4	13.27
106 × 3-3080-P	1.02	2	138.5	11.05	0.95	1	139.9	8.70	150.8	10.57

**Table 14:** Approach #D, FEA and experiment results of the cross-section set 104 × 2 CHS pin-ended columns.

Specimen [38]	Material Set SCP				Material Set TP				Experiment	
	$\bar{\lambda }$	cl.	*N_u,FE1_* (kN)	*ω_u,FE1_* (mm)	$\bar{\lambda }$	cl.	*N_u,FE2_* (kN)	*ω_u,FE2_* (mm)	*N_u,exp_* (kN)	*ω_u,exp_* (mm)
104 × 2-550-P	0.21	3	228.7	2.29	0.20	3	224.4	3.15	241.1	−2.21
104 × 2-750-P	0.28	3	218.6	2.33	0.28	3	211.0	3.54	232.1	−1.95
104 × 2-950-P	0.35	3	206.4	3.48	0.35	3	197.3	4.61	204.2	5.17
104 × 2-1150-P	0.43	3	195.6	4.68	0.42	3	186.5	5.63	180.8	6.24
104 × 2-1650-P	0.62	3	163.2	10.42	0.61	3	158.1	9.10	154.8	10.50
104 × 2-2150-P	0.79	4	132.0	14.05	0.79	3	132.0	9.92	126.4	15.19
104 × 2-2650-P	0.97	4	108.2	15.65	0.97	4	111.1	12.01	109.0	19.40
104 × 2-3080-P	1.14	4	95.15	18.32	1.13	3	98.91	14.70	89.7	22.81

**Table 15:** Comparison of approaches #A and #D. Summary of the average *N_u,FE_*/*N_u,exp_* and *ω_u,FE_*/*ω_u,exp_* values for varying material properties, based on the results of the cross-section sets 106 × 3 and 104 × 2 (Table 8, Table 9, Table 13, and Table 14).

Approach of this Study	Approach #A (Only Two Sets)		Approach #D (Only Two Sets)	
Material parameter value set	TP	SCP	TP	SCP
Local imperfection amplitude	0	0	0	0
Global imperfection amplitude	*ω*_0_ + *e*_0_	*ω*_0_ + *e*_0_	*ω*_0_ + *e*_0_	*ω*_0_ + *e*_0_
Mean *N_u,FE_*/*N_u,exp_*	0.964	1.001	0.962	0.999
COV *N_u,FE_*/*N_u,exp_*	0.065	0.051	0.065	0.051
Mean *ω_u,FE_*/*ω_u,exp_*	0.489	0.611	0.493	0.613
COV *ω_u,FE_*/*ω_u,exp_*	1.753	1.188	1.728	1.173

**Table 16:** Approach #A; Summary of the average *N_u,FE_*/*N_u,exp_* and *ω_u,FE_*/*ω_u,exp_* values for varying material properties.

Study	Approach #A, This Study		C. Buchanan et al. [38]	
Material parameter value set	TP	SCP	TP	SCP
Local imperfection amplitude	0	0	*t*/10	*t*/100
Global imperfection amplitude	*ω*_0_ + *e*_0_	*ω*_0_ + *e*_0_	*ω*_0_ + *e*_0_	*ω*_0_ + *e*_0_
Mean *N_u,FE_*/*N_u,exp_*	1.010	0.998	1.024	1.013
COV *N_u,FE_*/*N_u,exp_*	0.073	0.050	0.081	0.046
Mean *ω_u,FE_*/*ω_u,exp_*	0.627	0.660	0.612	0.708
COV *ω_u,FE_*/*ω_u,exp_*	1.006	0.825	0.920	0.834

## Data Availability

Data sharing not applicable.
